# Multi-omics data of gastric cancer cell lines

**DOI:** 10.1186/s12863-023-01122-9

**Published:** 2023-04-20

**Authors:** Eun-Hye Seo, Yun-Jae Shin, Hee-Jin Kim, Jeong-Hwan Kim, Yong Sung Kim, Seon-Young Kim

**Affiliations:** 1 Korea Bioinformation Center, Korea Research Institute of Bioscience and Biotechnology, Daejeon, Republic of Korea; 2 Department of Functional Genomics, Korea University of Science and Technology, Daejeon, Republic of Korea; 3 Aging Convergence Research Center, Korea Research Institute of Bioscience and Biotechnology, Daejeon, Republic of Korea; 4 Personalized Genomic Medicine Research Center, Korea Research Institute of Bioscience and Biotechnology, Daejeon, Republic of Korea

**Keywords:** Gastric cancer, Gastric cancer cell lines, RNA sequencing, Exome sequencing, ChIP-sequencing

## Abstract

**Objectives:**

Gastric cancer (GC) is the fourth most common cancer worldwide, with the highest incidence and mortality regardless of sex. Despite technological advances in diagnosing and treating gastric cancer, GC still has high incidence and mortality rates. Therefore, continuous research is needed to overcome GC. In various studies, cell lines are used to find and verify the cause of specific diseases. Large-scale genomic studies such as ENCODE and Roadmap epigenomic projects provide multiomics data from various organisms and samples. However, few multi-omics data for gastric tissues and cell lines have been generated. Therefore, we performed RNA-seq, Exome-seq, and ChIP-seq with several gastric cell lines to generate a multi-omics data set in gastric cancer.

**Data description:**

Multiomic data, such as RNA-seq, Exome-seq, and ChIP-seq, were produced in gastric cancer and normal cell lines. RNA-seq data were generated from nine GC and one normal gastric cell line, mapped to a human reference genome (hg38) using the STAR alignment tool, and quantified with HTseq. Exome sequence data were produced in nine GC and two normal gastric lines. Sequenced reads were mapped and processed using BWA-MEM and GATK, variants were called by stralka2, and annotation was performed using ANNOVAR. Finally, for the ChIP-seq, nine GC cell lines and four GC cell lines were used in two experimental sets; chip-seq was performed to confirm changes in H3K4me3 and H3K27me3. Data was mapped to human reference hg38 with BWA-MEM, and peak calling and annotation were performed using the Homer tool. Since these data provide multi-omics data for GC cell lines, it will be useful for researchers who use the GC cell lines to study.

## Objective

According to Global Cancer Statistics, gastric cancer (GC) is the fourth most common cancer worldwide, with the highest incidence and mortality regardless of gender, excluding female breast cancer, and more than 700,000 deaths annually from gastric cancer [1]. Over the past few decades, although there have been many advances in the discovery of biomarkers for early diagnosis of GC and surgical, chemical and immunological methods for treatment through many studies, gastric cancer still has high incidence and mortality rates. Therefore, continuous research is needed to diagnose and treat GC [2, 3].

Cell lines are a population of cells that represent the functions of specific tissues and can be cultured stably for a long period. They are the most convenient tools used for biology research. Cell lines provide many advantages, such as easy cultivation and use, low cost, and the absence of ethical concerns associated with the use of animal and human tissues [4, 5].

In various studies, cell lines are used to find and verify the cause of specific diseases. The data described in this work were initially produced to confirm the genomic and epigenomic landscape of GC cell lines. However, it has not been published because the data set contains samples of low depth or insufficient quality. Large-scale genomic studies such as ENCODE [6] and Roadmap epigenomic projects [7] provide researchers with insight into the mechanism of gene regulation by producing multi-omics data from various organisms and samples. However, only limited data is available for cell line data; particularly, there are no data on gastric cell lines. Therefore, this data set contains various omics data, including RNA-seq, Exome-seq, and ChIP-seq, which can be used in various ways to study GC. We hope that this resource will be useful to GC researchers.

Data Description.

Fourteen GC cell lines, including SNU001, SNU005, SNU016, SNU216, SNU520, SNU620, SNU638, SNU668, SNU719, AGS, MKN1, MKN45, MKN74, KATO III, and the normal gastric cell line Hs738, were obtained from the Korean Cell Line Bank (https://cellbank.snu.ac.kr) and American Type Culture Collection (https://www.atcc.org). A normal gastric cell line, HFE145, was previously established by H. Ashktorab and D. T. Smoot [8, 9]. Cell line sources and data lists are summarized in Data File 1.

Total RNA was extracted from eight GC cell lines and one normal gastric cell line using the RNeasy Mini kit (Qiagen). An RNA sequencing library was prepared using the TruSeq RNA sample prep kit (Illumina), and sequencing was performed using the Nextseq500 platform (illumina) to generate 75-bp paired-end reads. The sequenced reads were mapped to a human reference genome (hg38) using the STAR alignment tool (version 2.7.8a), and gene expression was quantified with the HTseq. The mapping results of the RNA sequencing data are shown in Data File 2. Each sample produced from 20 million to a maximum of 35 million reads. The mapped reads ranged from 17 million to 29 million, except for the normal cell line, Hs738. In Hs738, 13 million reads were produced, 11 million reads were uniquely mapped, and the mapping rate was about 90%.

Total genomic DNA was extracted from eight GC cell lines and two normal gastric lines using the DNeasy blood and Tissue kit (Qiagen). The sequencing library was prepared using the Roche NimbleGen SeqCap EZ Exome Library SR (Roche). Then, sequencing was performed using the Hiseq X Ten platform (Illumina) to generate 150 bp paired-end reads. Sequencing reads were mapped to the human hg38 reference genome using the BWA-MEM algorithm (v0.7.12-r1039) [10]. The resulting SAM files were transformed into BAM files using the samtools. Duplicate reads were eliminated using Picard MarkDuplicates, and the BAM files were processed using RealignerTargetCreator (GATK) to create the target intervals file for the IndelRealigner (GATK) to target local realignment. Local realignment of reads was performed to correct misalignments due to indels. BaseRecalibrator (GATK) was used to identify systematic errors in base quality scores exported from the sequencer and compute a recalibration model to adjust quality scores accordingly. PrintReads was performed as the final GATK analysis to produce re-calibrated merged output bam files sorted in coordinate order [11]. The variants were called using strelka2 [12]. Finally, the resulting set was annotated using ANNOVAR [13].

The Exome sequencing data include mapping rate, genome coverage, scores of the mapping quality scores, and duplicate reads, as shown in Data file 3. Each sample produced about 43 million to a maximum of 73 million reads, except for HFE145, which produced about 26 million reads, and showed an average mapping rate of more than 99% and a mapping quality score of more than 29. Duplicate reads were about 34%, and the genome coverage was about 1.7 on average. The human exome represents less than 2% of the genome; therefore, that coverage is sufficient to detect copy number variation (CNV) and structural variation in the genome [14]. The average number of variants per sample was 36,733 (from 24,299 to 46,191). The spectrum of base substitution of samples is shown in Data File 4. Each cell line showed a different base substitution ratio, but the C > A transversion was generally the highest, followed by the T > C transition.

The chromatin immunoprecipitation (ChIP) assay was performed with nine GC cells in the ChIP set1 and four GC cells in the ChIP set2 for ChIP sequence analysis following a protocol from the Myers lab (http://hudsonalpha.org/myers-lab/protocols) with modifications. Specifically, cells were fixed with 1% formaldehyde, lysed, and sonicated using a Covaris M220 (Covaris). For ChIP-seq analysis, the sonicated lysates for GC cells were used by dividing the same amount into three tubes and 10% input. Normal Rabbit IgG (2 µg, Sigma-Aldrich), anti-trimethyl-Histone H3 (Lys4) (2 µg, Sigma-Aldrich), and anti-trimethyl-Histone H3 (Lys27) (2 µg, Sigma-Aldrich) were prebound to 20 µl Dynabeads coupled with protein A or protein G (Invitrogen). Genomic libraries were prepared at 250 to 400 bp sizes with input and immunoprecipitated fragments using the TruSeq ChIP Sample Prep kit (Illumina). The ChIP-Seq library was sequenced using NextSeq_500 (Illumina), generating 76-bp single reads. The sequenced reads of the ChIP-seq were aligned with the human reference genome (hg38) using BWA-MEM (v0.7.12-r1039) [10]. ChIP peaks were called using a hypergeometric optimization of Motif EnRichment (Homer, version 4.11) [15] and annotated using the Homer annotatePeaks module.

ChIP-sequencing data have two experiment sets, and quality and quantity are summarized in Data File 5 and Data file 6. In the ChIP set1, each sample showed about 24 million to a maximum of 69 million reads, an average mapping rate of more than 96%, and a mapping quality score of more than 23.77. The duplication rate ranged from 3 to 71.17%, and the genome coverage ranged from about 0.5 to 1.5. In the ChIP set2, each sample showed about 16 million to a maximum of 41 million reads, an average mapping rate of more than 95% and a mapping quality score of more than 25.16. The duplication rate ranged from 3 to 20.30%, and the genome coverage ranged from about 0.3 to 0.9. The approximate IP efficiency is summarized in Data file 7. Data file 8 shows total tags in peaks / total tags after Homer peak calling, which shows a wide range for each sample but was generally more than 10% in the H3K4me3 immunoprecipitated samples. The immunoprecipitated H3K27me3 samples were generally less than 2%. The excessively high IP efficiency is due to the high duplication levels (Table 1).

**Table 1:** Overview of data files/data sets

Label	Name of data file/data set	File types (file extension)	Data repository and identifier (DOI or accession number)
Data file 1	Sources and Data Lists of Cell Lines	Dataset (.xlxs)	Figshare (10.6084/m9.figshare.20453322) [16]
Data file 2	Summary Statistics of RNA-seq Alignment	Dataset (.xlxs)	Figshare (10.6084/m9.figshare.20453802) [17]
Data file 3	Quality and quantity of the Exome-seq data	Dataset (.xlxs)	Figshare (10.6084/m9.figshare.20454027) [18]
Data file 4	Exome_TiTv_plot	Portable Document Format file (.pdf)	Figshare (10.6084/m9.figshare.20454138) [19]
Data file 5	Quality and quantity of the sequencing data_ChIP_set1	Dataset (.xlxs)	Figshare (10.6084/m9.figshare.20454267) [20]
Data file 6	Quality and quantity of the sequencing data_ChIP_set2	Dataset (.xlxs)	Figshare (10.6084/m9.figshare.20454279) [21]
Data file 7	ChIP seq IP efficiency ChIP_set1	Dataset (.xlxs)	Figshare (10.6084/m9.figshare.20454294) [22]
Data file 8	ChIP seq IP efficiency ChIP_set2	Dataset (.xlxs)	Figshare (10.6084/m9.figshare.20454381) [23]
Data set 1	GC RNA seq	Fastq file (fastq.gz)	Korea Nucleotide Archive (Accession No.: KRA2200860) [24] Sequence Read Archive (Accession No.: PRJNA892250) [28]
Data set 2	GC Exome seq	Fastq file (fastq.gz)	Korea Nucleotide Archive (Accession No.: KRA2200861) [25] Sequence Read Archive (Accession No.: PRJNA892250) [28]
Data set 3	GC ChIP seq_set1	Fastq file (fastq.gz)	Korea Nucleotide Archive (Accession No.: KRA2200862) [26] Sequence Read Archive (Accession No.: PRJNA892250) [28]
Data set 4	GC RNA seq_set2	Fastq file (fastq.gz)	Korea Nucleotide Archive (Accession No.: KRA2200863) [27] Sequence Read Archive (Accession No.: PRJNA892250) [28]

## Limitations

Since the data set contained samples of low depth or insufficient quality, observation of the genomic and epigenomic landscape of whole GC cell lines is challenging. Only four GC cell lines have a data set of three types (RNA-Seq, Exome-Seq, and ChIP-Seq), and the other cell lines miss one or two data types.

## Data Availability

The data file 1–8 described in this Data Note can be freely and openly accessed on FigShare (https://figshare.com/) [16–23]. The data set 1–4 were deposited in the Korea Nucleotide Archive (KoNA, https://kobic.re.kr/kona) with open accession ID KRA2200860, KRA2200861, KRA2200862, and KRA2200863 [24–27] and the NCBI Sequence Read Archive (SRA, https://www.ncbi.nlm.nih.gov/sra) with open accession ID PRJNA892250 [28].

## Abbreviations

GC: Gastric cancer of the GC
GATK: Genome Analysis Tool Kit
CNV: Copy number variation
ChIP: Chromatin immunoprecipitation
Homer: Hypergeometric Optimization of Motif Enrichment
